# A Snapshot of the Hepatic Transcriptome: *Ad Libitum* Alcohol Intake Suppresses Expression of Cholesterol Synthesis Genes in Alcohol-Preferring (P) Rats

**DOI:** 10.1371/journal.pone.0110501

**Published:** 2014-12-26

**Authors:** Jonathon D. Klein, Jeremy B. Sherrill, Gabriella M. Morello, Phillip J. San Miguel, Zhenming Ding, Suthat Liangpunsakul, Tiebing Liang, William M. Muir, Lawrence Lumeng, Amy C. Lossie

**Affiliations:** 1 Purdue University Interdisciplinary Life Sciences Graduate Program, Purdue University, West Lafayette, IN, United States of America; 2 Department of Animal Science, Purdue University, West Lafayette, IN, United States of America; 3 Core Facility, Purdue University, West Lafayette, IN, United States of America; 4 Department of Horticulture and Landscape Architecture, Purdue University, West Lafayette, IN, United States of America; 5 Department of Psychiatry, Indiana University School of Medicine, Indianapolis, IN, United States of America; 6 Department of Medicine, Indiana University School of Medicine, Indianapolis, IN, United States of America; INRA, France

## Abstract

Research is uncovering the genetic and biochemical effects of consuming large quantities of alcohol. One prime example is the J- or U-shaped relationship between the levels of alcohol consumption and the risk of atherosclerotic cardiovascular disease. Moderate alcohol consumption in humans (about 30 g ethanol/d) is associated with reduced risk of coronary heart disease, while abstinence and heavier alcohol intake is linked to increased risk. However, the hepatic consequences of moderate alcohol drinking are largely unknown. Previous data from alcohol-preferring (P) rats showed that chronic consumption does not produce significant hepatic steatosis in this well-established model. Therefore, free-choice alcohol drinking in P rats may mimic low risk or nonhazardous drinking in humans, and chronic exposure in P animals can illuminate the molecular underpinnings of free-choice drinking in the liver. To address this gap, we captured the global, steady-state liver transcriptome following a 23 week free-choice, moderate alcohol consumption regimen (∼7.43 g ethanol/kg/day) in inbred alcohol-preferring (iP10a) rats. Chronic consumption led to down-regulation of nine genes in the cholesterol biosynthesis pathway, including *HMG-CoA reductase*, the rate-limiting step for cholesterol synthesis. These findings corroborate our phenotypic analyses, which indicate that this paradigm produced animals whose hepatic triglyceride levels, cholesterol levels and liver histology were indistinguishable from controls. These findings explain, at least in part, the J- or U-shaped relationship between cardiovascular risk and alcohol intake, and provide outstanding candidates for future studies aimed at understanding the mechanisms that underlie the salutary cardiovascular benefits of chronic low risk and nonhazardous alcohol intake.

## Introduction

The liver, the primary site of alcohol metabolism, is susceptible to many of the pharmacological ramifications of drinking, and rodent models of human consumption have been critical for elucidating these consequences. The ethanol-containing Lieber-DeCarli [1] and the Tsukamoto-French intragastric liquid diets [2] are widely accepted methods that produce maximal liver injury by force-feeding rodents with ethanol. The Lieber-DeCarli approach delivers 12–18 g ethanol/kg/day (d), with mean blood alcohol concentrations (BACs) reaching 290–380 mg/dL [3], while the Tsukamoto-French method achieves BACs as high as 500 mg/dL. The liver develops many injuries in these forced ethanol administrations, including: severe accumulation of macro- and microvesicular fat, major structural and biochemical changes in organelles, activation of stellate cells, evidence of early fibrosis and apoptosis.

However, it is becoming increasingly clear that not all pharmacological effects of ethanol on liver processes are linearly related to ethanol dose and BAC levels. One prime example is the J- or U-shaped relationship between increasing alcohol consumption and the risk of atherosclerotic cardiovascular disease (not cardiomyopathy). Several studies [4]–[6] and a large meta-analysis report [7] concluded that light to moderate alcohol consumption in humans (i.e. 30 g ethanol/d) is associated with a reduced risk of coronary heart disease and ischemic stroke, while heavier alcohol intake is linked to increased risk. The underlying mechanisms responsible for this well-documented J- or U-shaped relationship remain unsolved. Suggested mechanisms include a complex interaction between HDL and LDL cholesterol transfer dynamics in producing atherosclerosis, the potential deleterious effects of ethanol on the biology of atherosclerotic plaques, as well as oxidative stress, [8], [9]. Moderate alcohol consumption is inversely correlated with metabolic syndrome and diabetes mellitus in obese individuals, diabetes mellitus [10], as well as vascular dementia in the aging population [11].

To better model moderate levels of consumption, it is necessary to develop a different paradigm. Most outbred rodents exhibit an innate aversion to voluntary ethanol drinking and do not ingest enough ethanol to raise BACs to pharmacological levels. One solution is to use the selectively bred alcohol-preferring (P) rat. The P/NP (preferring/non-preferring) rat lines were raised by bidirectional selective breeding for divergent alcohol drinking preferences when fed an *ad libitum* diet and given a free choice of drinking 10% (v/v) ethanol or water [12], [13]. P rats must drink>5 g ethanol/kg/d whereas NP rats must drink <1 g ethanol/kg/d. P rats typically drink 6.9±0.2 g of ethanol/kg/d during the dark cycle and attain BACs of 50–90 mg/dL within three hours. Behavioral studies indicate that they accomplish this by drinking in several short spurts (1–4 bouts; <6 minutes/bout) of>1 g of ethanol/kg/bout throughout the dark cycle. Accordingly, P rats satisfy all the criteria proposed for an animal model of alcoholism and meet many of the DSM-IV behavioral criteria for diagnosing alcohol abuse and alcoholism [13]–[15]. With the foreknowledge that between-species comparisons can be controversial, we suggest herein that the extent of free-choice alcohol drinking in P rats, with respect to the liver, ostensibly models low risk or nonhazardous alcohol drinking in humans.

Most hepatic transcriptome studies focus on high levels of forced ethanol consumption that produced maximum liver steatosis and injury [16]–[18]. Although epidemiological evidence links light drinking with a protective effect, there is a dearth of knowledge about the effects of free-choice drinking on the liver in P animals. Therefore, the goals of this study were to: 1. Determine the effect of nonhazardous alcohol quantities produced by chronic, free-choice drinking on intrahepatic lipid accumulation and 2. Identify the hepatic gene networks that are altered during long-term, nonhazardous, moderate alcohol usage. To address these goals, we documented hepatic cholesterol and lipid levels and profiled the hepatic transcriptome using RNA-Sequencing (RNA-Seq) following long-term free-choice alcohol drinking. We report here that daily, voluntary consumption of pharmacologically relevant levels of alcohol for 23 weeks alters expression of many genes in the liver without causing hepatic steatosis. In congruence with the phenotypic data, gene ontology analysis indicates that genes involved in cholesterol synthesis are down-regulated in alcohol consuming animals. This study, with a primary focus on the liver transcriptome, is consistent with the involvement of the cholesterol pathway in the association between moderate alcohol intake and reduced risk for heart disease and stroke.

## Results

Several epidemiological studies link moderate alcohol intake with reduced risk for coronary heart disease and ischemic stroke. Although, the molecular mechanisms remain largely unsolved, top candidates are cholesterol dynamics, oxidative stress and other unidentified biological effects of ethanol on atherosclerotic lesions. To better understand the impact of moderate alcohol consumption on the liver, we examined the effects of moderate, free-choice alcohol consumption on hepatic triglyceride and cholesterol levels, as well as the hepatic transcriptome in animals consuming *ad libitum* levels of alcohol for 23 weeks. To dampen the between-animal variance, we used the inbred alcohol-preferring P rat strain, iP10a.

### Lack of steatosis and normal lipid profiles in both groups

**Figure 1:**
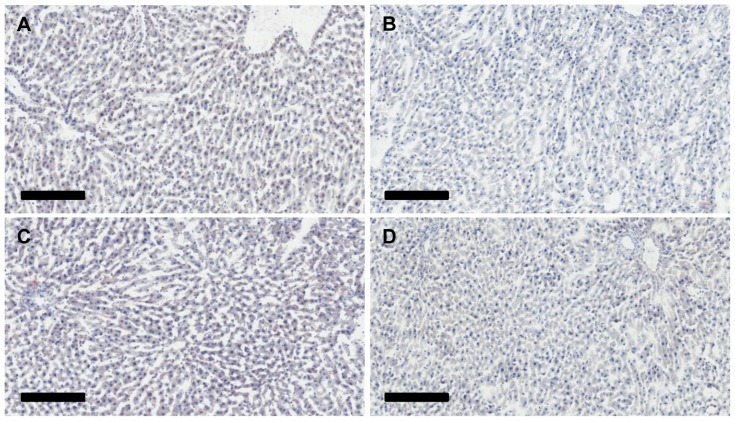
Histological analysis of livers. Liver sections were stained using Oil Red O and Harris hematoxylin. (**A**) and (**B**) Representative images of H_2_O animal livers. (**C**) and (**D**) Representative images of EtOH livers. There is no evidence of hepatic steatosis in the livers of EtOH animals following 23 weeks of ethanol exposure. The scale bar is 200 µm.

Chronic, high dose ethanol feeding in mice leads to intrahepatic fat (both triglycerides and cholesterol) accumulation. In contrast, we see no histological (Fig. 1; H&E and Oil Red O stains) or biochemical (hepatic triglycerides and cholesterol) changes in P rats consuming ethanol *ad libitum*. The livers of both groups (EtOH, n = 6; Control, n = 6) are virtually identical and show no identifiable lipid globules, indicating that this level of alcohol usage did not result in histological steatosis in the liver. Analysis of total hepatic triglycerides and cholesterol in 6 EtOH livers and 5 controls further confirms this finding. Triglyceride (Control group: 856±92 mg triglycerides/mg protein, EtOH group: 863±48 mg triglycerides/mg protein, p = 0.886) and cholesterol (Control group: 451±27 mg cholesterol/mg protein, EtOH group: 469±7.4 mg cholesterol/mg protein, p = 0.219) levels were indistinguishable between the two groups.

### Misregulation of genes important for cholesterol biosynthesis and formation of the cytoskeleton

By design, this study emulates human alcohol consumption behaviors, creating an ideal opportunity to catalog the global hepatic changes in gene expression that occur in liver following chronic, moderate alcohol usage. Understanding these alterations are paramount, as the liver metabolizes the majority of ingested alcohol through a series of steps that oxidize ethanol into acetaldehyde and then into acetate [19], [20]. To document the full complement of altered transcripts, we compared the hepatic transcriptome of six animals that had free-choice access to alcohol and six control individuals. All samples were pooled and paired-end sequenced in a single lane, with individual samples identified by a unique barcode. Samples averaged 13.1 million reads, and yielded an average of 1.2 Gb of sequence per sample. Approximately 67% of reads mapped to our reference genome, 97% of which mapped uniquely. Both groups shared 19,410 transcripts; an additional 1,456 transcripts were only detected in the water controls, while 1,546 transcripts were unique to EtOH animals.

**Figure 2:**
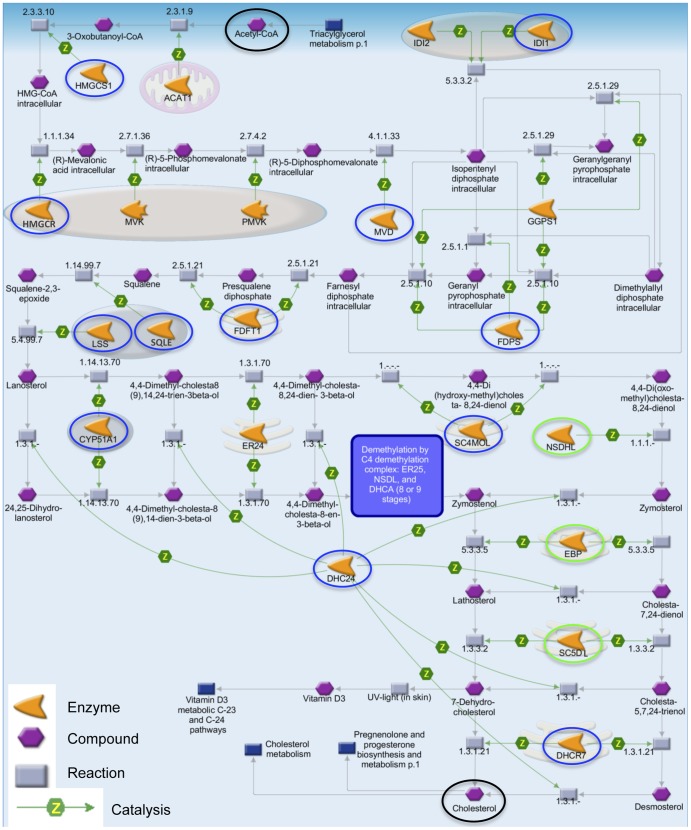
The cholesterol biosynthesis pathway. Moderate alcohol consumption decreased expression of 15 genes throughout the cholesterol biosynthesis pathway. Blue circles denote genes with a fold change greater than −1.3, while green denote those less than −1.3. All enzymes (orange arrowheads) produced from these genes catalyzed (green arrows labeled Z) their respective chemical reactions (grey boxes) on their target compounds (purple hexagons). The first compound, Acetyl-CoA, and the last compound, Cholesterol, are denoted by black circles (top and bottom of figure, respectively). The figure also displays the cellular localization of the enzymes: the entire figure is found in the cytoplasm, pink ovals represent peroxisomes (e.g. IDI1), blue ovals are lysosomes (e.g. LSS), and pink stacks represent endoplasmic reticulum (e.g. FDFT1). *Srebf1* and *Srebf2*, transcription factors that activate many of these genes, were both down-regulated (not displayed). Nine of these results were confirmed by qRT-PCR (Fig. 3A). Image modified from Thomson Reuter's MetaCore.

Alcohol-exposure led to altered expression of 259 transcripts from 258 genes, with a false discovery rate (FDR) of 5% (S1 Table). The majority of these transcripts (215) were down-regulated, while 44 were up-regulated in ethanol-treated animals. We determined the potential functional consequences of these altered transcription levels by gene ontology analysis using the MetaCore platform from Thomson Reuters. To achieve greater biological significance, we restricted our input to genes demonstrating a fold-change greater than ±1.3. This reduced the list to 94 transcripts (74 down-regulated and 20 up-regulated, Table 1) from 16 significantly altered pathways (Table 2). The cholesterol biosynthesis pathway (Fig. 2) contained 12 misregulated genes (with a±1.3 fold cut-off) and was the most prominent pathway (p = 3.635e-14). Other pathways included: regulatory aspects of cellular metabolism, with a bias for lipid and ketone metabolism; and components of the cytoskeleton, particularly microfilaments and microtubules.

**Table 1:** Genes with ±1.3 or greater fold change after chronic ethanol treatment.

Symbol	Gene Name	Fold Change	Corrected p-value
*Aacs*	Acetoacetyl-CoA synthetase	−2.4913	0.0090
*Abcc4*	ATP-binding cassette, subfamily C (CFTR/MRP), member 4	−1.7972	0.0287
*Acat3*	Acetyl-Coenzyme A acetyltransferase 3	−1.9462	0.0113
*Acsm2*	Acyl-CoA synthetase medium-chain family member 2A	−1.7458	0.0311
*Actg1*	Actin, gamma 1	−1.3990	0.0106
*Aldh1a7*	Aldehyde dehydrogenase family 1, subfamily A7	−8.0352	0.0226
*Ankrd44*	Ankyrin repeat domain 44	1.4922	0.0442
*Apex1*	APEX nuclease (multifunctional DNA repair enzyme) 1	−1.4432	0.0262
*Arhgap29*	Rho GTPase activating protein 29	1.3020	0.0415
*Arl5b*	ADP-ribosylation factor-like 5B	1.3029	0.0324
*Basp1*	Brain abundant, membrane attached signal protein 1	−1.6695	0.0131
*Bckdk*	Branched chain ketoacid dehydrogenase kinase	−1.3679	0.0056
*Bcl3*	B-cell CLL/lymphoma 3	−1.4100	0.0393
*Cbs*	Cystathionine beta synthase	−1.3411	0.0415
*Creb3l1*	cAMP responsive element binding protein 3-like 1	−1.3710	0.0324
*Csad*	Cysteine sulfinic acid decarboxylase	−2.3927	0.0463
*Csmd1*	CUB and Sushi multiple domains 1	−2.3761	0.0157
*Cyp51*	Cytochrome P450, family 51	−1.5255	0.0056
*D3ZT51*		−1.4147	0.0415
*Dguok*	Deoxyguanosine kinase	*	0.0009
*Dhcr24*	24-dehydrocholesterol reductase	−1.3771	0.0391
*Dhcr7*	7-dehydrocholesterol reductase	−1.9364	0.0307
*Dph1*	DPH1 homolog (S. cerevisiae)	−1.4021	0.0392
*Efhc1*	EF-hand domain (C-terminal) containing 1	^#^	0.0055
*Ephb1*	Eph receptor B1	^#^	0.0262
*Fam73b*	Family with sequence similarity 73, member B	−1.3103	0.0452
*Fdft1*	Farnesyl diphosphate farnesyl transferase 1	−1.5468	0.0105
*Fdps*	Farnesyl diphosphate synthase	−1.4441	0.0095
*Gck*	Glucokinase	−2.1995	0.0080
*Gclc*	Glutamate-cysteine ligase, catalytic subunit	−1.3119	0.0182
*Gm6484*	Predicted gene 6484	−1.8603	0.0195
*Gpatch3*	G patch domain containing 3	−1.3652	0.0476
*Gsta3*	Glutathione S-transferase alpha 3	−1.5341	0.0415
*Hes6*	Hairy and enhancer of split 6 (Drosophila)	−1.5687	0.0416
*Hip1r*	Huntingtin interacting protein 1 related	−1.3085	0.0080
*Hmgcr*	3-hydroxy-3-methylglutaryl-CoA reductase	−1.6268	0.0324
*Hmgcs1*	3-hydroxy-3-methylglutaryl-CoA synthase 1 (soluble)	−2.0623	0.0064
*Hsd17b7*	Hydroxysteroid (17-beta) dehydrogenase 7	−1.5221	0.0480
*Idi1*	Isopentenyl-diphosphate delta isomerase 1	−2.0524	0.0056
*Irs2*	Insulin receptor substrate 2	1.6998	0.0415
*Itgal*	Integrin, alpha L	1.3163	0.0339
*Kif26b*	Kinesin family member 26B	−3.0480	0.0350
*Klf9*	Kruppel-like factor 9	1.4918	0.0195
*LOC100359951*		−1.3843	0.0415
*LOC100361376*		−1.4853	0.0389
*LOC494499*		−1.5858	0.0441
*LOC682888*		−1.4953	0.0172
*Lss*	Lanosterol synthase (2,3-oxidosqualene-lanosterol cyclase)	−1.5008	0.0022
*Me1*	Malic enzyme 1, NADP(+)-dependent, cytosolic	−1.7325	0.0308
*Mid1ip1*	MID1 interacting protein 1 (gastrulation specific G12 homolog (zebrafish))	−1.5504	0.0262
*Mrps18b*	Mitochondrial ribosomal protein S18B	−1.3708	0.0262
*Mrps2*	Mitochondrial ribosomal protein S2	−1.3651	0.0009
*Mrps34*	Mitochondrial ribosomal protein S34	−1.3264	0.0365
*Mvd*	Mevalonate (diphospho) decarboxylase	−2.0150	0.0195
*Nabp1*	Oligonucleotide/oligosaccharide-binding fold containing 2A	−1.3573	0.0255
*Ndufaf4*	NADH dehydrogenase (ubiquinone) 1 alpha subcomplex, assembly factor 4	−1.3623	0.0106
*Oat*	Ornithine aminotransferase	1.3563	0.0324
*Olr1587*	Olfactory receptor 1587	7.9442	0.0415
*Pam*	Peptidylglycine alpha-amidating monooxygenase	1.3639	0.0324
*Pck1*	Phosphoenolpyruvate carboxykinase 1 (soluble)	1.7312	0.0090
*Pcsk9*	Proprotein convertase subtilisin/kexin type 9	−1.3723	0.0324
*Pinx1*	PIN2/TERF1 interacting, telomerase inhibitor 1	−1.4089	0.0375
*Pir*	Pirin (iron-binding nuclear protein)	−1.9381	0.0370
*Ppp1r3b*	Protein phosphatase 1, regulatory subunit 3B	−1.3082	0.0195
*Psat1*	Phosphoserine aminotransferase 1	−1.7933	0.0450
*Psme3*	Proteasome (prosome, macropain) activator subunit 3	1.6157	0.0171
*Ptcd2*	Pentatricopeptide repeat domain 2	−1.3706	0.0056
*Ptpre*	Protein tyrosine phosphatase, receptor type, E	*	0.0056
*Rrm2*	Ribonucleotide reductase M2	2.4855	0.0434
*Sc4mol*	Methylsterol monooxygenase 1	−1.9263	0.0056
*Scly*	Selenocysteine lyase	−1.3971	0.0113
*Scn1b*	Sodium channel, voltage-gated, type I, beta	−1.4340	0.0056
*Sds*	Serine dehydratase	2.4717	0.0441
*Sds*	Serine dehydratase	4.7055	0.0114
*Sema3d*	Sema domain, immunoglobulin domain (Ig), short basic domain, secreted, (semaphorin) 3D	*	0.0044
*Sert1 (AF077195)*	Sertoli cell protein 1	−10.2588	0.0415
*Sfrs2*	Serine/arginine-rich splicing factor 2	−1.3264	0.0169
*Sfrs3*	Serine/arginine-rich splicing factor 3	−1.4130	0.0072
*Sqle*	Squalene epoxidase	−2.1774	0.0056
*Srebf1*	Sterol regulatory element binding transcription factor 1	−1.5895	0.0420
*Tcp11l2*	T-complex 11 (mouse) like 2	1.3396	0.0499
*Tlr13*	Toll-like receptor 13	1.6008	0.0480
*Tmem97*	Transmembrane protein 97	−1.4464	0.0142
*Tsku*	Tsukushi	−2.1529	0.0182
*Tuba1c*	Tubulin, alpha 1C	−1.3522	0.0476
*Tuba4a*	Tubulin, alpha 4A	−1.6102	0.0098
*Tubb2a*	Tubulin, beta 2A class IIa	−1.6534	0.0113
*Tubb4b*	Tubulin, beta 4B class IVb	−1.3448	0.0216
*Upp2*	Uridine phosphorylase 2	15.3404	0.0202
*Wdr18*	WD repeat domain 18	−1.3181	0.0182
*Yars*	Tyrosyl-tRNA synthetase	−1.4044	0.0098
*Zbtb43*	Zinc finger and BTB domain containing 43	1.3794	0.0337
*Zfand2a*	Zinc finger, AN1-type domain 2A	−1.3342	0.0407
*Zmynd19*	Zinc finger, MYND-type containing 19	−1.3301	0.0339

* - *Gene was solely expressed in water control.* ^#^ - *Gene was solely expressed in ethanol treatment.*

**Table 2:** GeneGO pathway map analysis.

#	Pathway	Genes	p-value
1	Cholesterol Biosynthesis	*Cyp51, Dhcr24, Dhcr7, Fdft1, Fdps, Hmgcr, Hmgcs1, Idi1, Lss, Mvd, Sc4mol, Sqle*	3.99E-14
2	Cytoskeleton remodeling_Neurofilaments	*Actg1, Tuba1c, Tuba4a, Tubb2a, Tubb4b*	1.08E-05
3	Cell adhesion_Gap Junctions	*Actg1, Tuba1c, Tuba4a, Tubb2a, Tubb4b*	2.28E-05
4	Cytoskeleton remodeling_Keratin filaments	*Actg1, Tuba1c, Tuba4a, Tubb2a, Tubb4b*	4.78E-05
5	Regulation of metabolism_Bile acids regulation of glucose and lipid metabolism via FXR	*Me1, Pck1, Srebf1*	5.34E-05
6	wtCFTR and Δ508 traffic/Clathrin coated vesicles formation (norm and CF)	*Actg1, Hip1r*	1.58E-04
7	Development_Role of IL-8 in angiogenesis	*Actg1, Hmgcr, Srebf1*	3.14E-04
8	Butanoate metabolism	*Aacs, Hmgcs1, Acat3*	4.31E-04
9	Mitochondrial ketone bodies biosynthesis and metabolism	*Aacs, Hmgcs1, Acat3*	4.61E-04
10	Development_Slit-Robo signaling	*Actg1, Tuba1c, Tuba4a, Tubb2a, Tubb4b*	6.33E-04
11	Cytoskeleton remodeling_Reverse signaling by ephrin B	*Actg1, Tuba1c, Tuba4a, Tubb2a, Tubb4b*	6.98E-04
12	Cell cycle_Role of Nek in cell cycle regulation	*Tuba1c, Tuba4a, Tubb2a, Tubb4b*	7.67E-04
13	Regulation of lipid metabolism_Regulation of lipid metabolism via LXR, NF-Y and SREBP	*Cyp51, Srebf1*	1.27E-03
14	Regulation of lipid metabolism_Insulin regulation of fatty acid metabolism	*Gck, Irs2, Srebf1*	1.59E-03
15	Transport_Macropinocytosis	*Actg1, Tuba1c, Tuba4a, Tubb2a, Tubb4b*	2.02E-03
16	Regulation of lipid metabolism_Regulation of fatty acid synthase activity in hepatocytes	*Srebf1*	5.11E-03

The level of ethanol consumption seen in our animals had an extensive effect on the genes underlying the cholesterol biosynthesis pathway (Table 2; Fig. 2). In Fig. 2: orange arrowheads represent enzymes encoded by their respective genes; all enzymes in this pathway had a positive effect on their individual chemical reactions (grey boxes), in other words catalyzing the reaction (represented by green arrows labeled Z); chemical compounds are represented by purple hexagons; blue boxes display other pathways linked via various metabolites. All genes from the pathway elicited in our RNA-Seq study were down-regulated and are circled; blue circles are genes with fold changes greater than −1.3 fold, and green circles are those with a fold change less than −1.3.

The entirety of the cholesterol biosynthesis pathway occurs in the cytoplasm and begins with acetyl-CoA (black circle at top center). First, the mitochondrial enzyme acetyl-CoA acetyltransferase (*Acat1*, not altered in our study) catalyzes the formation of acetoacetyl-CoA from two acetyl-CoA molecules. Next, hydroxymethylglutaryl-CoA (HMG-CoA) synthase 1 (*Hmgcs1*, −2.06 fold change) catalyzes the conversion of acetoacetyl-CoA and acetyl-CoA to HMG-CoA. Then, HMG-CoA reductase (*Hmgcr*, −1.63 fold change) catalyzes the formation of NADP+ and mevalonic acid from HMG-CoA, NA(P)H and H+. *Hmgcs1* and *Hmgcr* are both considered rate-limiting steps in cholesterol synthesis [21]. Following this, mevalonate kinase (*Mvk*, not altered) activates an ATP-dependent phosphorylation of mevalonic acid to mevalonate 5-phosphate, which is phosphorylated by phosphomevalonate kinase (*Pmvk*, not altered) to form 5-diphosphomevalonate. *Hmgcr*, *Mvk*, and *Pmvk* are all peroxisomal enzymes (pink ovals). Diphosphomevalonate decarboxylase (*Mvd*, −2 fold change) decarboxylates 5-diphosphomevalonate to isopentenyl pyrophosphate (IPP). At this point the pathway splits into parallel options. In the first branch, IPP delta isomerase 1 (*Idi1*, −2.05 fold change) and IPP delta isomerase 2 (*Idi2*, not altered) convert IPP to dimethylallyl diphosphate (DMAPP). *Idi1* and *Idi2* are both peroxisomal (blue-pink ovals). DMAPP is condensed to geranyl diphosphate and to farnesyl diphosphate. Both reactions are catalyzed by either farnesyl diphosphate synthase (*Fdps*, −1.44 fold change) or geranylgeranyl diphosphate synthase 1 (*Ggps1*, not altered). Alternatively, IPP can be converted to geranylgeranyl diphosphate by *Ggps1*. A third option is the conversion of IPP to geranyl diphosphate and then to farnesyl diphosphate by *Fdps*. Lastly, IPP can be converted directly to farnesyl diphosphate by *Ggps1*.

All reactions culminate in the production of farnesyl disphosphate, which is then converted to presqualene diphosphate followed by squalene; both reactions are catalyzed by farnesyl diphosphate farnesyl transferase 1 (*Fdft1*, −1.55 fold change). *Fdft1* is located in the endoplasmic reticulum. From there squalene epoxidase (*Sqle*, −2.18 fold change) and lanosterol synthase (*Lss*, −1.50 fold change), both lysosomal enzymes, convert squalene to squalene-2-3-epoxide and then to lanosterol. A series of three steps involving *Cyp51* a cytochrome P450 gene (−1.53 fold change), transmembrane 7 superfamily member 2 (*Tm7sf2*, not altered) and methylsterol monooxygenase 1 (*Sc4mol*, −1.93 fold change) leads to the sequential conversion of lanosterol to 4,4-di(oxomethyl)cholesta-8,24-dienol. Following that, NAD(P) dependent steroid dehydrogenase-like (*Nsdhl*, −1.18 fold change), emopamil binding protein (sterol isomerase) (*Ebp*, −1.23 fold change) and sterol-C5-desaturase (*Sc5dl*, −1.25 fold change) produce 7-dehydrodesmosterol from 4,4-di(oxomethyl)cholesta-8,24-dienol. Then, 7-dehydrocholesterol reductase (*Dhcr7*, −1.94 fold change) and 24-dehydrocholesterol reductase (*Dhcr24*, −1.38 fold change) remove the C(7–8) double bond and delta-24 double bond, respectively, to produce cholesterol (black circle, bottom center). *Dhcr24* may reduce the double bond in many of the cholesterol intermediates beginning with lanosterol, creating a variety of alternative steps. However, these alternatives are catalyzed by the same enzymes in the same order (lower half of Fig. 2).

Our RNA-seq studies demonstrated that ethanol exposure down-regulated 15 of the 21 genes in the cholesterol biosynthesis pathway, including *Hmgcr* and *Hmgcs1*. Three of these genes, *Nsdhl*, *Ebp*, and *Sc5dl*, were identified in our RNA-Seq data but were excluded from MetaCore analysis because they failed to make the cutoff of −1.3 X alteration in expression, leaving a total of 12 genes in the cholesterol biosynthesis pathway for further study. We re-analyzed these 12 genes quantitatively using TaqMan chemistry to confirm ethanol-induced changes in expression. We chose *18S* rRNA for our endogenous control, as it displays the least amount of variance in all stages of alcoholic liver disease in humans (compared to *GAPDH*, *ACTB*, and *SFRS4* [22]). For each gene, we performed qRT-PCR on 5 or 6 biological replicates. One technical replicate was included for each biological sample to control for pipetting errors. We selected genes based on gene ontology analysis, i.e. cholesterol metabolism and cellular infrastructure (Table 2).

**Figure 3:**
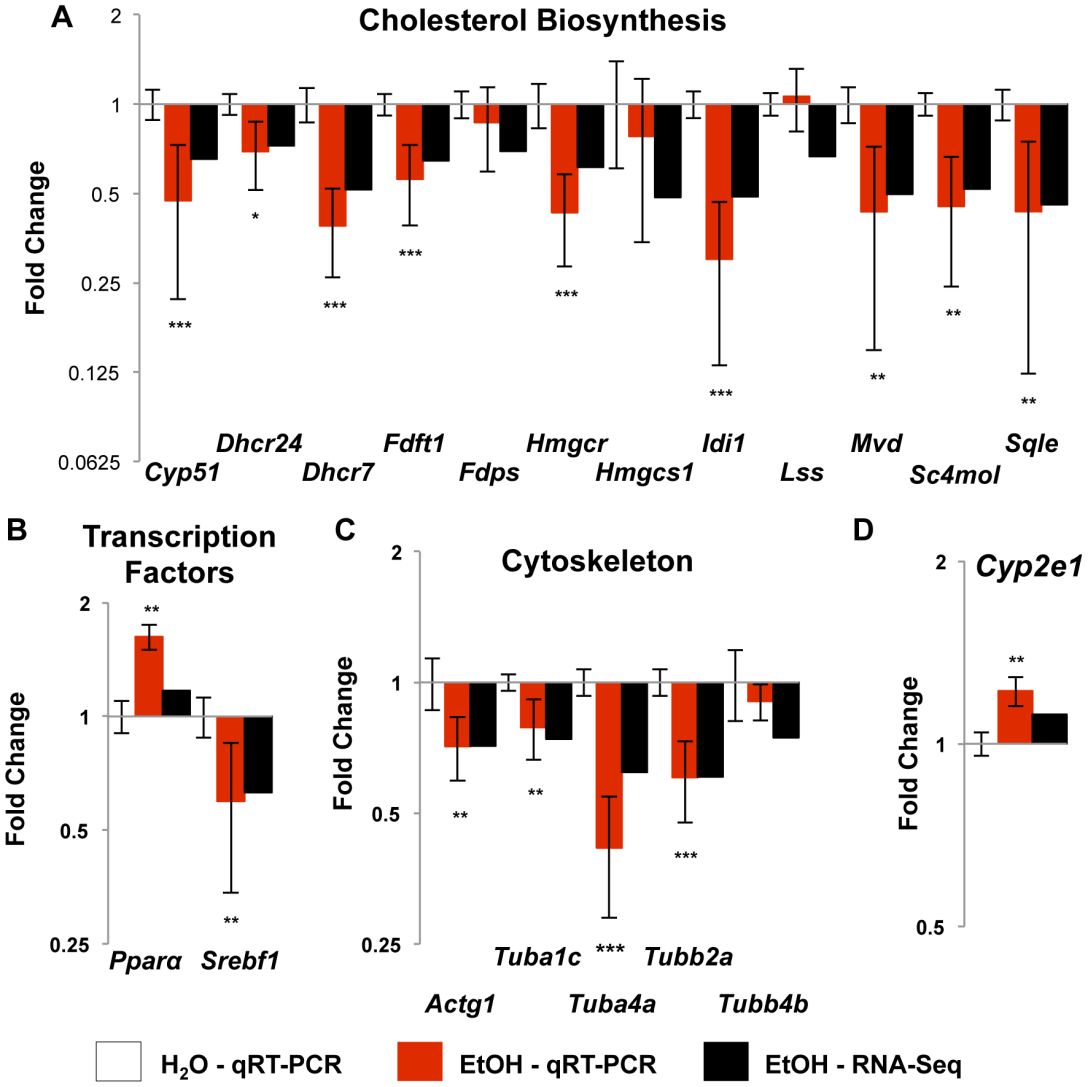
Confirmation of altered expression by qRT-PCR in functional pathway genes. Taqman-based qRT-PCR was used to confirm the changes in gene expression. These graphs display the fold change (±SEM) of the candidate genes by qRT-PCR (red bars) and RNA-Seq (black bars) analysis for comparison. Numbers are relative to the water treated control rats (set at 1.0). (**A**) Nine of twelve genes in the cholesterol biosynthesis pathway were confirmed. (**B**) *Pparα*, a transcription factor that activates genes for cholesterol oxidation was not significantly altered by RNA-Seq analysis, but was significantly up-regulated by qRT-PCR. *Srebf1*, a transcriptioin factor that activates many genes in the synthesis pathway, was significantly decreased in both analyses. (**C**) Four cytoskeleton subunit genes were suppressed by RNA-Seq and qRT-PCR. (**D**) *Cyp2e1*, a gene induced by chronic high levels of alcohol intake that mediates various alcohol-induced injuries was not induced when measured by RNA-Seq analysis, but was significantly up-regulated by qRT-PCR. * - p-value <0.05, ** - p-value <0.005, *** - p-value <0.0001.

These studies confirmed that nine members of the cholesterol biosynthesis pathway were down-regulated in qRT-PCR studies (Fig. 3A). The three exceptions were *Fdps*, which catalyzes the production of geranyl pyrophosphate and farnesyl pyrophosphate from isopentenyl pyrophosphate and dimethylallyl pyrophosphate, *Hmgcs1*, which synthesizes acetyl-CoA, acetoacetyl-CoA, and H_2_O, and *Lss*, which forms lanosterol. None of these were significantly altered by qRT-PCR. We then examined two transcription factors that aid in the regulation of cholesterol homeostasis (Fig. 3B): *Srebf1*, which activates many cholesterol biosynthesis genes [21]; and *Pparα*, which activates genes orchestrating lipid oxidation [23]. We confirmed that *Srebf1* was significantly decreased (p = 0.0009). Although *Pparα* was not identified in our RNA-Seq data, it was significantly up-regulated by qRT-PCR (p = 0.0024).

We also confirmed altered expression of five actin and tubulin genes (*Actg1, Tuba1c, Tuba4a, Tubb2a, Tubb4b*), which are key components of seven additional pathways (Table 2 # 2–4, 10–12, 15). *Actg1* is a cytoplasmic actin and a subunit of microfilaments involved in internal motility. Alpha and beta tubulins (*Tuba1c*, *Tuba4a*, *Tubb2a*, *Tubb4b*) are the major constituents of microtubules, which provide the scaffold for organelle organization and protein trafficking. Four of these (*Actg1, Tuba1c, Tuba4a, Tubb2a*), showed altered expression by both RNA-Seq and qRT-PCR (Fig. 3C).

It is well documented that the gene product of *Cytochrome P450 2E1* (*Cyp2e1*) is responsive to high levels of ethanol consumption, resulting in increased generation of reactive oxygen species (ROS) [24], [25]. High levels of ROS increase oxidative stress in the cell, which likely promotes the various hepatic injuries seen following high levels of alcohol consumption [26], [27]. Although we did not examine ROS, we found a significant (1.22 fold; p = 0.0044, Fig. 3D) increase in expression of *Cyp2e1* by qRT-PCR.

**Figure 4:**
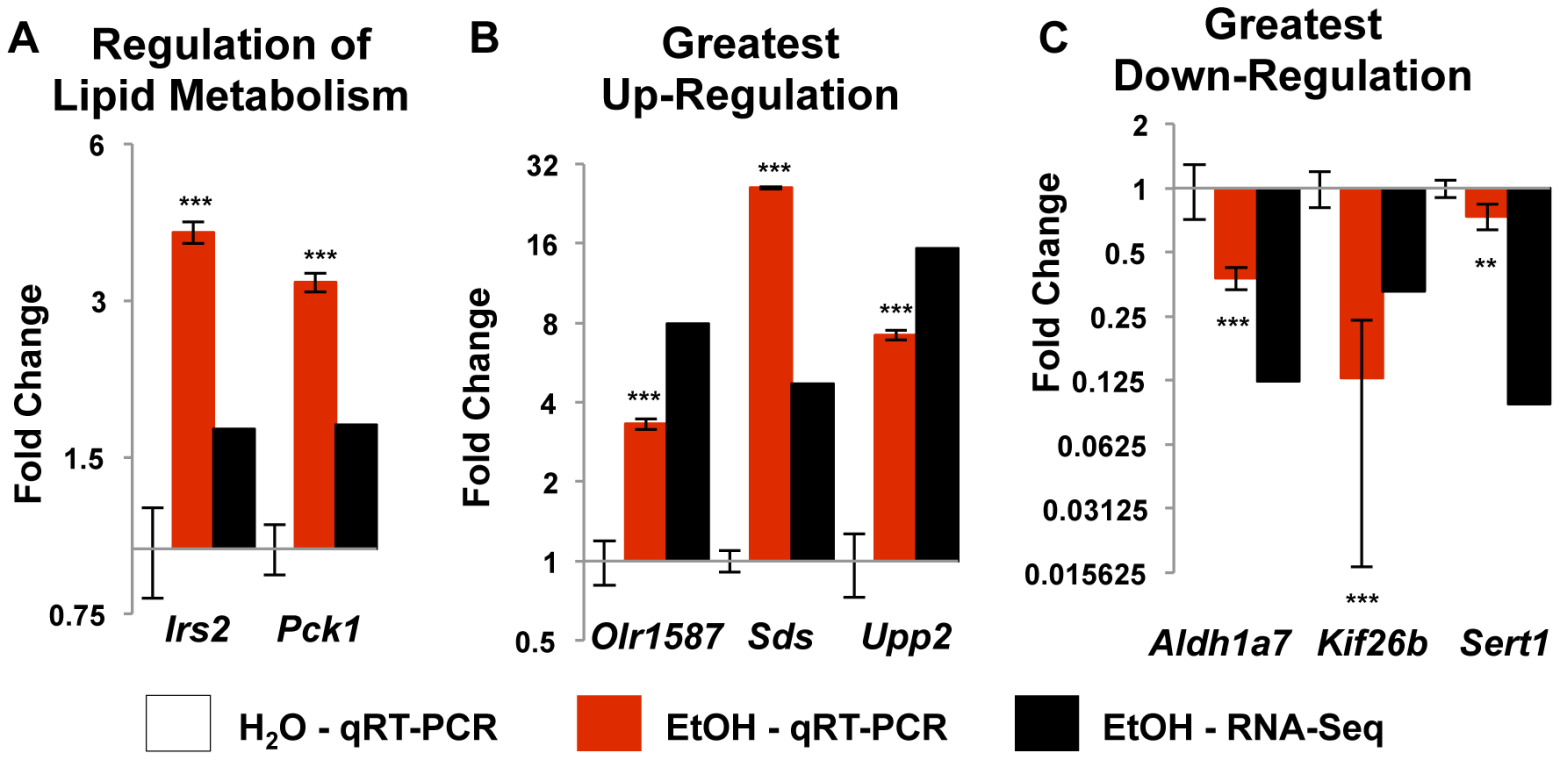
Altered expression by qRT-PCR. (**A**) *Irs2* and *Pck1*, involved in the regulation of lipid metabolism, were up-regulated by RNA-Seq analysis and confirmed by qRT-PCR. (**B**) and (**C**) confirm the genes with the greatest up-regulation and down-regulation, respectively. The direction of the change was confirmed for all 6 genes, though not necessarily to the same degree. ** - p-value <0.005, *** - p-value <0.0001.

To ensure there was not a technical bias in the data, as most genes were down-regulated in the alcohol group, we confirmed two up-regulated genes, *Irs2* and *Pck1*. These genes are involved in regulating glucose and lipid metabolism (Table 3) and were up-regulated in both analyses (Fig. 4A). Lastly, we used qRT-PCR to confirm the genes with the greatest fold change in both directions; *Olr1587*, *Sds*, and *Upp2* were increased, while *Aldh1a7*, *Kif26b*, and *Sert1* were decreased (Fig. 4B, C) by both measurements.

**Table 3:** TaqMan assays used for qRT-PCR confirmation.

Gene	Name	Sequence ID
*Actg1*	Actin, gamma 1	NM_001127449.1
*Aldh1a7*	Aldehyde dehydrogenase family 1, subfamily A7	NM_017272.15
*Cyp2e1*	Cytochrome P450, family 2, subfamily e, polypeptide 1	NM_031543.1
*Cyp51*	Cytochrome P450, family 51	NM_037073.1
*Dhcr24*	24-dehydrocholesterol reductase	NM_001080148.1
*Dhcr7*	7-dehydrocholesterol reductase	NM_022389.2
*Fdft1*	Farnesyl diphosphate farnesyl transferase 1	NM_019238.2
*Fdps*	Farnesyl diphosphate synthase	NM_031840.1
*Hmgcr*	3-hydroxy-3-methylglutaryl-CoA reductase	NM_013134.2
*Hmgcs1*	3-hydroxy-3-methylglutaryl-Coenzyme A synthase (soluble)	NM_017268.1
*Idi1*	Isopentenyl-diphosphate delta isomerase 1	NM_053539.1
*Irs2*	Insulin receptor substrate 2	NM_001168633.1
*Kif26b*	Kinesin family member 26B	NM_001109079.2
*Lss*	Lanosterol synthase (2,3-oxidosqualene-lanosterol cyclase)	NM_031049.1
*Mvd*	Mevalonate (diphospho) decarboxylase	NM_031062.1
*Olr1587*	Olfactory receptor 1587	NM_001000913.1
*Pck1*	Phosphoenolpyruvate carboxykinase 1 (soluble)	NM_198780.3
*Ppara*	Peroxisome proliferator-activated receptor alpha	NM_013196.1
*Sc4mol*	Methylsterol monooxygenase 1	NM_080886.1
*Sds*	Serine dehydratase	NM_053962.3
*Sert1 (AF077195)*	Sertoli cell protein 1	AF077195.1
*Sqle*	Squalene epoxidase	NM_017136.2
*Srebf1*	Sterol regulatory element binding transcription factor 1	XM_213329.5
*Tuba1c*	Tubulin, alpha 1C	NM_001011995.1
*Tuba4a*	Tubulin, alpha 4A	NM_001007004.1
*Tubb2a*	Tubulin, beta 2A class IIa	NM_001109119.1
*Tubb4b*	Tubulin, beta 4B class IVb	NM_199094.1
*Upp2*	Uridine phosphorylase 2	NM_001106481.1
*18S*	18S ribosomal RNA	X03205.1

## Discussion

To the best of our knowledge, this is the first study to use RNA-Seq to analyze the hepatic transcriptome following chronic alcohol exposure. RNA-Seq overcomes many of the limitations of hybridization-based methods. NGS data has lower noise, provides accuracy over a wide range of expression levels [28], and removes the dependence on species with well annotated genomes [29]. We validated the use of RNA-Seq via qRT-PCR confirmation of 28 genes in multiple genetic and biochemical pathways.

Free-choice alcohol consumption closely reflects alcohol use behavior in humans [12], [30]. A caveat of voluntary consumption is that most outbred rodents consume only low, and therefore pharmacologically irrelevant levels of alcohol. We avoided this issue by using inbred iP10a rats as test subjects and providing animals free access to multiple concentrations of ethanol solution [31], as providing multiple alcohol concentrations to P rats increases their ethanol intake [32]. We also monitored consumption, measured absolutely by grams of ethanol/kg/day, to ensure that all animals consumed relevant quantities. Animals voluntarily consumed an average of 7.4 g ethanol/kg/d during the first 12 weeks. This level of intake produced BACs ranging from 50–90 mg/dL. Consumption during the subsequent 11 weeks was estimated based on prior data. We did not use a pair-feeding strategy to equalize the caloric intake for the EtOH or H_2_O groups, as P rats self-regulate their calorie intake by reducing chow intake to compensate for the calories from the ethanol solution (L. Lumeng, unpublished data). We collected body weights measurements throughout the first 12 weeks of the study (data not shown), and found no differences between the controls and alcohol-treated animals at any timepoint. Although this does not preclude differences in intake, it suggests that the animals receiving free-choice alcohol were not consuming extra calories. As such, it is unlikely that differences in caloric intake are responsible for these results.

Our data indicate that 23 weeks of free-choice alcohol consumption significantly represses expression of genes involved in cholesterol biosynthesis and cytoskeleton remodeling. Although altered expression does not necessarily reflect a change in protein level, Deaciuc and colleagues [16] found that changes in protein levels reflected altered expression for 4 out of 5 genes following four weeks of forced ethanol exposure.

### Hepatic Cholesterol and Triglycerides

High levels of alcohol intake (BACs≥300 mg/dL) can cause severe fatty liver (steatosis) following acute and chronic alcohol exposure in mice [18], [33]. Microarray studies conducted on animals following forced ethanol exposure (to achieve high BACs and an inordinate degree of steatosis) reported altered expression patterns that likely promote increased cholesterol synthesis in the liver [16]–[18]. However, the present study, which was designed to assess the hepatic transcriptome of animals undergoing a nonhazardous drinking paradigm, detected no steatosis, normal hepatic cholesterol and triglyceride levels and a suppression of steady-state mRNA levels of nine genes in the cholesterol synthesis pathway (Tables 2.3 and 2.4), including HMG-CoA reductase (*Hmgcr*) and sterol regulatory element binding transcription factor 1 (*Srebf1*). *Hmgcr* is the rate-limiting enzyme for cholesterol synthesis activity in the liver and *Srebf1* is the activating transcription factor for many cholesterol synthesis genes [21].

These findings agree with Lakshmanan and Veech [34], who determined that feeding ethanol in drinking water for 21 days caused a 29% decrease in *Hmgcr*, presumably due to BACs that are much lower than those observed with forced ethanol feeding. Long-term studies of the consequences of free-choice drinking in P and iP rats indicates that there are minimal phenotypic liver insults at six months [35], further supporting our findings.

It is beyond the scope of this paper to address exactly how down-regulation of hepatic cholesterol synthesis genes can have a salutary effect on cardiovascular risks. However, it is well established that down-regulation of hepatic cholesterol synthesis genes, as can be achieved by the use of statins, can consistently suppress plasma LDL and raise circulating HDL. High circulating HDL levels are anti-atherogenic because of “reverse cholesterol transport” [36]. Additionally, moderate ethanol intake is associated with increases in paraoxonase 1 (PON1) activity (plasma and liver) and expression (liver) [37]. PON1 is an HDL-associated anti-atherogenic enzyme that inhibits the oxidation of LDL, mediates the conversion of oxidized LDL to inactive products, and inhibits cholesterol uptake by macrophages in the intima of atheromas [38]. Our results led us to propose that moderate and nonhazardous alcohol drinking could provide the same benefits as a statin, and thereby administer a significant cardioprotective benefit. In addition to down-regulation of *Hmgcr*, moderate alcohol drinking in the iP10a rats also led to decreased expression of eight other genes that are important in hepatic cholesterol synthesis. Together, these data led to the supposition that the protective effects (as visualized by the J- or U-shaped curves) of long-term intake of nonhazardous amounts of alcohol could be due to decreased hepatic biosynthesis of cholesterol, thereby suppressing circulating LDL levels and raising plasma HDLs.

### Alcohol Metabolism

One puzzling finding is that only one of the potential alcohol metabolism genes (*Aldh1a7*) was upregulated in this study. Most alcohol is metabolized sequentially from ethanol to acetaldehyde to acetate by two classes of enzymes: alcohol dehydrogenases (ADH enzymes) and aldehyde dehydrogenases (ALDH enzymes) [19]. ADH enzymes function in the cytosol, converting ethanol to acetaldehyde through a NAD+ dependent mechanism. Approximately 85 to 90% of alcohol is metabolized by ADH enzymes to acetaldehyde in the cytosol [19]. Secondary hepatic alcohol metabolizing enzymes include catalase, which processes ∼2% of hepatic alcohol into acetaldehyde in the peroxisome fraction and a handful of cytochrome P450 enzymes (CYP2E1, CYP1A2 and CYP3A4) that oxidize ∼10% of total ingested alcohol to acetaldehyde within the endoplasmic reticulum.

We saw no changes in expression of any of the *Adh* genes, but did see increased expression of *Cyp2e1* by qRT-PCR, indicating that chronic alcohol consumption could be causing a shift from cytosolic metabolism to oxidation within the endoplasmic reticulum. Usually, *Cyp2e1* is induced by heavy ethanol consumption [39]–[41]; in our study, the animals followed a moderate, chronic alcohol intake. How chronic ethanol administration leads to induction of *Cyp2e1* remains controversial. Elevated levels of *Cyp2e1* mRNA have been reported in some studies [42], while others have reported little or no induction of *Cyp2e1* mRNA [43], [44] following a chronic alcohol paradigm. In P animals, CYP2E1 studies show elevated protein and enzyme activity levels following six months of free-choice drinking, with consumption levels at 8 to 9 g/kg/d [35]. These conditions closely mimic our study, and suggest that alcohol can be metabolized by CYP2E1 in P rats.

One byproduct of CYP2E1 metabolism is the production of reactive oxygen species [19], [24], [25], [45]), which promote various cellular and DNA injuries [26], [27]. qRT-PCR studies indicated that chronic free-choice drinking increased mRNA levels of *Cyp2e1* by 22%. Future studies aimed at determining if increased oxidative stress accompanied the upregulation of *Cyp2e1* will be important to better understand the pathophysiology associated with chronic, but moderate alcohol consumption.

Acetaldehyde, the first oxidized product of alcohol metabolism, damages multiple components of the cell, including DNA, where it induces intrastrand cross-linkages [46]. Acetaldehyde is oxidized to acetate primarily by ALDH2 protein isoforms, called the Class II ALDH enzymes [47]. We saw no upregulation of any of the ALDH genes, including the Class II ALDH2 isoforms. This, along with the modest increase (22%) in *Cyp2e1* expression, indicates that alcohol metabolism overall had not changed, but the method of oxidation of ethanol to acetaldehyde may have shifted slightly due to chronic ingestion.

Although we saw no evidence for a marked change in ethanol breakdown, we detected a marked decrease in the steady-state levels of *Aldh1b7* (an ∼8 fold reduction in alcohol-treated animals), a member of the Class I acetaldehyde dehydrogenase family. The primary target of most of the Class I enzymes, including *Aldh1b7*, is retinol, not acetyaldehyde [48]. Therefore, decreased levels of *Aldh1b7* mRNA may indicate that other oxidation reactions, but not alcohol metabolism, are disrupted.

### Cellular Cytoskeleton

Five structural genes involved in formation and function of the cytoskeleton (*Actg1, Tuba1c*, *Tuba4a*, *Tubb2a*, *Tubb4b*) were downregulated in alcohol-treated animals. The cytoskeleton, which is composed of microfilaments, intermediate filaments and microtubules, provides a scaffold for cytoplasmic organization, cellular trafficking and protein migration/motility. We found that chronic ethanol consumption significantly down-regulated *Actin*, *gamma 1* (*Actg1*) and several alpha and beta tubulins (*Tuba1c*, *Tuba4a*, *Tubb2a*, *Tubb4b*). ACTG1 is a subunit of microfilaments, while the tubulins are microtubule subunits. These findings add to current discrepancies in the field; altered expression levels of several tubulin genes have been reported in studies examining the effects of high doses of alcohol. For example, various cytoskeleton and extracellular matrix genes, including *Tubb2b*, were down-regulated in Park's study [49], in which a Lieber-DeCarli diet was administered for 7 weeks. This is in agreement with our findings. However, Deaciuc and colleagues [16] determined that *Tuba1* and *Tubb5* were up-regulated after 4 weeks of intragastric ethanol infusion, while Yoon *et al* [50] found no changes in the levels of α-tubulin following 8–17 weeks of a Lieber-DeCarli diet. These differences imply that the specific quantity and duration of alcohol exposure, not simply the presence of alcohol, are important determinants for affecting expression of cytoskeleton genes. Our data suggest that expression of several microfilament and microtubule subunit genes in hepatocytes are exquisitely sensitive (even with BACs in the range of 50 to 90 mg/dL) to the effects of ethanol.

### Alterations in mRNA Steady State levels

Since only 17 of the 94 transcripts in this study showed large changes in gene expression (>2 fold differences), we sought to gain a deeper understanding of the genes outside of the major pathways that were most affected by chronic alcohol consumption. We focused on *Upp2* (15.3 fold induction), *Orl1587* (7.9 fold induction, *Sds* (4.7 fold induction), *Sert1* (10.3 fold reduction) and *Kif26b* (3.0 fold reduction). We confirmed alterations in gene expression in all of these transcripts. We then looked for phenotypes that could be important for our study using The Phenotype-Genotype Integrator (PheGenI) [51] and PhenoGen Informatics [52]. PheGenI merges NHGRI genome-wide association study (GWAS) catalog data with databases housed at the National Center for Biotechnology Information (NCBI), including Gene, dbGaP, OMIM, GTEx and dbSNP. PheGenI can be accessed from: http://www.ncbi.nlm.nih.gov/gap/phegeni. PhenoGen Informatics, a separate website for quantitative analysis of transcriptome data, is housed at the University of Colorado Denver; PhenoGen Website [Internet]. Aurora (CO): University of Colorado and Denver Health Sciences Center. PhenoGen Informatics, 2005 - [cited (7–2014)]. PhenoGen Informatics is available from http://phenogen.ucdenver.edu.

Using PheGenI, we found an eQTL overlapping *Upp2* (uridine phosphorylase 2) that is associated with several phenotypes, including cardiovascular disease, atherosclerosis, stroke and obesity. This case-cohort study entitled, “*Building on GWAS for NHLBI-diseases: the U.S. CHARGE consortium (CHARGE-S): FHS*” is a sub-study from the Framingham cohort and is deposited into dbGAP (Accession # phs000651.v3.p8; Database of Genotypes and Phenotypes. Bethesda, (MD): National Center for Biotechnology Information, National Library of Medicine) [53]. dbGAP can be accessed at: http://www.ncbi.nlm.nih.gov/sites/entrez?db=gap.

PheGenI analysis of *Kif26b* (Kinesin family member 26b) revealed that 50 different studies had identified eQTLs associated with this genomic region. Eleven of these studies found associations with cholesterol and/or lipoproteins, while twenty showed strong associations with coronary disease and/or atherosclerosis. Other common associations include cardiovascular disease, obesity, gout and stroke. Although *Olr1587* is an olfactory receptor that is usually detected in the olfactory bulb, several eQTLs (identified by PhenoGen Informatics analysis) overlap the region, including a 2 Mb eQTL associated with increased serum triglycerides and a 45 Mb eQTL associated with alcohol response. No associations were found with *Sds* (serine dehydratase) or *Sert1* (sertoli cell transcript 1, a putative rat transcript that has not been identified in mouse or human).

### Conclusions

Free-choice alcohol drinking in alcohol-preferring P rats simulates moderate, nonhazardous alcohol drinking in humans. We identified 94 genes with alterations in gene expression at a false discovery rate of 5%. The majority of these alterations showed less than a 2-fold change in expression, including most of the genes within the cholesterol biosynthesis pathway. This information was taken into consideration during MetaCore analysis. One explanation is that subtle changes in gene expression along a large pathway could produce amplified effects on cholesterol production. In support of this possibility, we saw no evidence of steatosis in the alcohol-treated animals. This is in stark contrast to other studies using much larger quantities of alcohol [18], [33] that reported high levels of steatosis and gene expression patterns consistent with increased hepatic cholesterol synthesis [16]–[18]. Since these animals were under selection for 30 generations prior to brother-sister matings to produce inbred lines [54], one could predict that reducing cholesterol production beyond a certain threshold could have deleterious effects during the selection process.

Three of the five genes outside of the cholesterol, lipid or cytoskeleton pathways showing large increases or decreases in expression are associated with eQTLs for cardiovascular disease, coronary disease atherosclerosis and stroke, phenotypes that are highly relevant for this study. It is possible that misregulation of these strong candidates, along with a downregulation of the cholesterol pathway, explains, at least in part, the well-documented J- or U-shaped relationship between alcohol exposure and cardiovascular risks seen in humans. Although these initial studies were conducted in the liver, further experimentation of the epigenetic and genetic consequences of free-choice drinking in other tissues (e.g. the brain) is important to understand the molecular underpinnings of the behavioral consequences of long-term, voluntary alcohol drinking.

## Materials and Methods

### Ethics Statement

All procedures and treatments on live animals were conducted in facilities approved by the American Association for the Accreditation of Laboratory Animals with approval from the Indiana University School of Medicine Animal Care and Use Committee, protocol #, and complied with the IUSM IACUC guidelines, protocol # 0000003015.

### Rat Strains, Animal Husbandry and Alcohol Treatment

Twelve adult females from the 65^th^ generation of the inbred, alcohol-preferring, iP10a rats were divided evenly between ethanol treatment (EtOH) and water control (H_2_O) groups. This inbred alcohol-preferring (iP) strain was raised by repeated brother X sister matings when the P line reached the S30 generation of selective breeding. Chronic alcohol exposure for the EtOH group was achieved by 23 weeks of continuous access to 0, 10, 20 and 30% ethanol (v/v) in their home cage. Ethanol consumption at each concentration was monitored three times per week, and body weight was measured weekly for the first 12 weeks. EtOH animals consumed a daily average of 7.43 g ethanol/kg in the first 12 weeks. BACs ranged from 50–90 mg/dL. Animals were housed in hanging wire mesh cages with access to food (Teklad Irradiated F-6 Rodent Diet), tap water, and alcohol solutions *ad libitum*. The rat room followed a 12∶12 light∶dark cycle.

### Tissue Procurement

To prevent a spike in stress response during sacrifice, animals were habituated to daily handling and the guillotine for two weeks prior to euthanasia. Immediately after sacrifice, livers were snap frozen in a bath of isopentane and dry ice. Tissue was stored at −80°C until RNA isolation. BACs were undetectable at the time of sacrifice.

### Hepatic Triglyceride and Cholesterol Analysis

We inspected Oil Red O and hematoxylin stained slides using Aperio's ImageScope (Vista, CA) to identify lipids. We used approximately 20 mg of liver tissue powder, prepared under liquid nitrogen, for quantifying triglyceride and cholesterol levels. Lipids were extracted using isopropanol. Hepatic triglycerides and cholesterol were measured using Wako L-type TG M and Cholesterol E (Cat #439–17501) assay kits, respectively (Wako Diagnostics, Richmond, VA).

### RNA Isolation

Total RNA was isolated from frozen liver samples using RNA Stat-60 (Amsbio LLC, Lake Forest, CA) following the manufacturer's directions and stored at −80°C. Integrity was checked using the Agilent 2100 Bioanalyzer (Agilent Technology, Santa Clara, CA). All samples showed an RNA integrity number above 8.5.

### RNA-Sequencing

The Illumina TruSeq RNA kit was used for cDNA library preparation, largely following the manufacturer's directions with modifications detailed below. Poly(A) RNA was isolated by annealing biotinylated oligo-dT to total RNA followed by two purification cycles using streptavidin-conjugated magnetic beads. RNA was fragmented for 4 minutes at 94°C to increase the population of desired sized RNA fragments in the 200–600 nt range. cDNA synthesis was performed using random hexamers. Fragment ends were repaired and 3′ ends were adenylated prior to adaptor ligation. Each adaptor was uniquely bar-coded to assign each read to its respective sample during data analysis. Adaptor ligated fragments were enriched following 10 cycles of PCR. Resulting amplicons were pooled and size selected using a PippinPrep (Sage Science, Beverly, MA) set to collect 320–600 bp molecules. The final cDNA library was titrated by qRT-PCR and sequenced in 101 nucleotide paired-end reads on an Illumina HiScanSQ system. RNA-seq data has been deposited into the Gene Expression Omnibus (GEO) Database at NCBI (Accession # GSE46669).

### RNA-Seq Data Analysis

Sequences were analyzed and mapped using CLCbio Genomics Workbench 4.9 software (Cambridge, MA). Reads were normalized by reads per kilobase per million mapped reads (RPKM) to standardize for gene length, as described in Mortazavi [28]. Values were transformed by square root to stabilize variances and renormalized a second time by quantile normalization to adjust for among sample variation. A standard t-test with pooled error term was applied to determine p values, which were then corrected for a false discovery rate (FDR) of 5%.

### Quantitative RT-PCR

We used the samples from the RNA-Seq experiment to confirm gene expression differences by qRT-PCR. Total RNA was treated with DNaseI using the Ambion Turbo DNA-free kit (Cat #AM1907) following the manufacturer's protocol (ABI, Foster City, CA). cDNA was synthesized using the SuperScript VILO cDNA Synthesis kit (Cat #11754050) following manufacturer's instructions (Invitrogen, Carlsbad, CA). Finally, qRT-PCR was performed using TaqMan gene expression assays (Table 1) from ABI (Foster City, CA) on an ABI Prism 7000 Sequence Detection System. Samples were normalized to the endogenous *18S* rRNA control, and expression was determined by relative quantification (fold change  = 2^−ΔΔCt^).

### Data Analysis for qRT-PCR

The experimental design was a completely randomized design with a one-way arrangement of treatments (water vs. ethanol consumption), which were applied to a total of 5 or 6 animals. Data were analyzed through the Generalized Linear Model (GLM) procedure on SAS and comparisons between treatments were done through the Fisher's Least Square Differences method.

## Supporting Information

S1 Table **Genes statistically altered by chronic ethanol treatment.** This table includes all genes with a corrected FDR of 5%.
(DOCX)
