# Supplementary material for: A Snapshot of the Hepatic Transcriptome: Ad Libitum Alcohol Intake Suppresses Expression of Cholesterol Synthesis Genes in Alcohol-Preferring (P) Rats
Source: PLoS One. 2014 Dec 26;9(12):e110501. doi: 10.1371/journal.pone.0110501 (PMC4277277; doi:10.1371/journal.pone.0110501)
Supplement: S1 Table — Genes statistically altered by chronic ethanol treatment. This table includes all genes with a corrected FDR of 5%. (DOCX) [file pone.0110501.s001.docx]

**Supplementary Tables**

| **Table S1: Genes Significantly Altered By Chronic Ethanol Treatment.** | | | |
| --- | --- | --- | --- |
| **Symbol** | **Gene Name** | **Fold Change** | **Corrected p-value** |
| *Aacs* | Acetoacetyl-CoA synthetase | -2.4913 | 0.0090 |
| *Aars* | Alanyl-tRNA synthetase | -1.2071 | 0.0044 |
| *Abcc4* | ATP-binding cassette, subfamily C (CFTR/MRP), member 4 | -1.7972 | 0.0287 |
| *Abhd4* | Abhydrolase domain containing 4 | -1.1574 | 0.0443 |
| *Acat3* | Acetyl-Coenzyme A acetyltransferase 3 | -1.9462 | 0.0113 |
| *Acp1* | Acid phosphatase 1, soluble | -1.1425 | 0.0363 |
| *Acsm2* | Acyl-CoA synthetase medium-chain family member 2A | -1.7458 | 0.0311 |
| *Actb* | Actin, beta | -1.1882 | 0.0115 |
| *Actg1* | Actin, gamma 1 | -1.3990 | 0.0106 |
| *Adrm1* | Adhesion regulating molecule 1 | -1.2310 | 0.0416 |
| *Aldh1a7* | Aldehyde dehydrogenase family 1, subfamily A7 | -8.0352 | 0.0226 |
| *Ammecr1l* | AMME chromosomal region gene 1-like | 1.2192 | 0.0212 |
| *Ankrd44* | Ankyrin repeat domain 44 | 1.4922 | 0.0442 |
| *Apex1* | APEX nuclease (multifunctional DNA repair enzyme) 1 | -1.4432 | 0.0262 |
| *Arhgap29* | Rho GTPase activating protein 29 | 1.3020 | 0.0415 |
| *Arl5b* | ADP-ribosylation factor-like 5B | 1.3029 | 0.0324 |
| *Arl6ip4* | ADP-ribosylation-like factor 6 interacting protein 4 | -1.1941 | 0.0324 |
| *Atad3a* | ATPase family, AAA domain containing 3A | -1.2676 | 0.0267 |
| *Atf4* | Activating transcription factor 4 (tax-responsive enhancer element B67) | -1.2873 | 0.0169 |
| *Atp6v0a1* | ATPase, H+ transporting, lysosomal V0 subunit A1 | -1.2045 | 0.0262 |
| *Basp1* | Brain abundant, membrane attached signal protein 1 | -1.6695 | 0.0131 |
| *Bckdk* | Branched chain ketoacid dehydrogenase kinase | -1.3679 | 0.0056 |
| *Bcl3* | B-cell CLL/lymphoma 3 | -1.4100 | 0.0393 |
| *Bloc1s1* | Biogenesis of lysosomal organelles complex-1, subunit 1 | -1.1606 | 0.0304 |
| *Cbs* | Cystathionine beta synthase | -1.3411 | 0.0415 |
| *Ccdc80* | Coiled-coil domain containing 80 | 1.1561 | 0.0452 |
| *Cct3* | Chaperonin containing Tcp1, subunit 3 (gamma) | -1.1765 | 0.0375 |
| *Cct6a* | Chaperonin containing Tcp1, subunit 6A (zeta 1) | -1.1934 | 0.0324 |
| *Cd2bp2* | Cd2 (cytoplasmic tail) binding protein 2 | -1.1232 | 0.0191 |
| *Cdo1* | Cysteine dioxygenase, type I | 1.1218 | 0.0196 |
| *Cgn* | Cingulin | -1.2091 | 0.0365 |
| *Chchd2* | Coiled-coil-helix-coiled-coil-helix domain containing 2 | -1.1276 | 0.0476 |
| *Chkb* | Choline kinase beta | -1.1110 | 0.0449 |
| *Chpf2* | Chondroitin polymerizing factor 2 | -1.2768 | 0.0416 |
| *Cnbp* | CCHC-type zinc finger, nucleic acid binding protein | -1.1709 | 0.0113 |
| *Cnp* | 2',3'-cyclic nucleotide 3' phosphodiesterase | -1.2279 | 0.0113 |
| *Col1a2* | Collagen, type I, alpha 2 | 1.2857 | 0.0480 |
| *Commd5* | COMM domain containing 5 | -1.1754 | 0.0253 |
| *Copz1* | Coatomer protein complex, subunit zeta 1 | -1.1157 | 0.0349 |
| *Cps1* | Carbamoyl-phosphate synthetase 1 | 1.2039 | 0.0432 |
| *Creb3l1* | cAMP responsive element binding protein 3-like 1 | -1.3710 | 0.0324 |
| *Csad* | Cysteine sulfinic acid decarboxylase | -2.3927 | 0.0463 |
| *Csmd1* | CUB and Sushi multiple domains 1 | -2.3761 | 0.0157 |
| *Cyb5* | Cytochrome b5 type A (microsomal) | -1.0810 | 0.0462 |
| *Cyb5b* | Cytochrome b5 type B (outer mitochondrial membrane) | -1.1711 | 0.0375 |
| *Cyp51* | Cytochrome P450, family 51 | -1.5255 | 0.0056 |
| *D10Wsu52e* | DNA segment, Chr 10, Wayne State University 52, expressed | -1.1704 | 0.0231 |
| *D3ZD79* |  | -1.1987 | 0.0195 |
| *D3ZEY5* |  | 1.2231 | 0.0454 |
| *D3ZHQ9* |  | 1.1940 | 0.0434 |
| *D3ZHW0* |  | -1.2309 | 0.0339 |
| *D3ZT51* |  | -1.4147 | 0.0415 |
| *D3ZWJ1* |  | -1.2973 | 0.0196 |
| *D4Wsu53e* | DNA segment, Chr 4, Wayne State University 53, expressed | 1.2351 | 0.0438 |
| *Ddi2* | DNA-damage inducible protein 2 | 1.2464 | 0.0308 |
| *Def8* | Differentially expressed in FDCP 8 homolog (mouse) | -1.1633 | 0.0470 |
| *Dguok* | Deoxyguanosine kinase | ***** | 0.0009 |
| *Dhcr24* | 24-dehydrocholesterol reductase | -1.3771 | 0.0391 |
| *Dhcr7* | 7-dehydrocholesterol reductase | -1.9364 | 0.0307 |
| *Dio1* | Deiodinase, iodothyronine, type I | -1.2093 | 0.0416 |
| *Dnajc11* | DnaJ (Hsp40) homolog, subfamily C, member 11 | -1.2131 | 0.0324 |
| *Dnajc12* | DnaJ (Hsp40) homolog, subfamily C, member 12 | -1.2994 | 0.0226 |
| *Dnpep* | Aspartyl aminopeptidase | -1.1375 | 0.0113 |
| *Dph1* | DPH1 homolog (S. cerevisiae) | -1.4021 | 0.0392 |
| *Eaf1* | ELL associated factor 1 | -1.1064 | 0.0416 |
| *Ebp* | Phenylalkylamine Ca2+ antagonist (emopamil) binding protein (sterol isomerase) | -1.2263 | 0.0311 |
| *Efhc1* | EF-hand domain (C-terminal) containing 1 | **^#^** | 0.0055 |
| *Egfr* | Epidermal growth factor receptor | 1.2895 | 0.0262 |
| *Eif2b5* | Eukaryotic translation initiation factor 2B, subunit 5 epsilon | -1.1444 | 0.0195 |
| *Eif2s1* | Eukaryotic translation initiation factor 2, subunit 1 alpha | -1.2063 | 0.0476 |
| *Eif3b* | Eukaryotic translation initiation factor 3, subunit B | -1.1902 | 0.0392 |
| *Eif3c* | Eukaryotic translation initiation factor 3, subunit C | -1.1440 | 0.0395 |
| *Eif4a1* | Eukaryotic translation initiation factor 4A1 | -1.1987 | 0.0226 |
| *Enthd2* | ENTH domain containing 2 | -1.2227 | 0.0273 |
| *Ephb1* | Eph receptor B1 | **^#^** | 0.0262 |
| *Exosc4* | Exosome component 4 | -1.2915 | 0.0462 |
| *Fam50a* | Family with sequence similarity 50, member A | -1.1593 | 0.0204 |
| *Fam73b* | Family with sequence similarity 73, member B | -1.3103 | 0.0452 |
| *Fam98a* | Family with sequence similarity 98, member A | -1.2027 | 0.0324 |
| *Farsb* | Phenylalanyl-tRNA synthetase, beta subunit | -1.2848 | 0.0375 |
| *Fdft1* | Farnesyl diphosphate farnesyl transferase 1 | -1.5468 | 0.0105 |
| *Fdps* | Farnesyl diphosphate synthase | -1.4441 | 0.0095 |
| *Fetub* | Fetuin B | -1.1565 | 0.0251 |
| *Fmo3* | Flavin containing monooxygenase 3 | 1.2902 | 0.0392 |
| *Folr2* | Folate receptor 2 (fetal) | 1.2818 | 0.0476 |
| *Fus* | Fused in sarcoma | -1.2353 | 0.0226 |
| *Gck* | Glucokinase | -2.1995 | 0.0080 |
| *Gclc* | Glutamate-cysteine ligase, catalytic subunit | -1.3119 | 0.0182 |
| *Ggnbp2* | Gametogenetin binding protein 2 | 1.1112 | 0.0416 |
| *Ghr* | Growth hormone receptor | -1.1706 | 0.0349 |
| *Gid8* | Bwk1 leukemia-related gene | -1.0731 | 0.0324 |
| *Glyr1* | Putative oxidoreductase GLYR1 | -1.1099 | 0.0349 |
| *Gm5617* | Predicted gene 5617 | -1.2876 | 0.0378 |
| *Gm6484* | Predicted gene 6484 | -1.8603 | 0.0195 |
| *Gorasp2* | Golgi reassembly stacking protein 2 | -1.1599 | 0.0080 |
| *Gpatch3* | G patch domain containing 3 | -1.3652 | 0.0476 |
| *Gsta3* | Glutathione S-transferase alpha 3 | -1.5341 | 0.0415 |
| *Gtf2h1* | General transcription factor IIH, polypeptide 1 | -1.1832 | 0.0195 |
| *Hes6* | Hairy and enhancer of split 6 (Drosophila) | -1.5687 | 0.0416 |
| *Hip1r* | Huntingtin interacting protein 1 related | -1.3085 | 0.0080 |
| *Hmgcr* | 3-hydroxy-3-methylglutaryl-CoA reductase | -1.6268 | 0.0324 |
| *Hmgcs1* | 3-hydroxy-3-methylglutaryl-CoA synthase 1 (soluble) | -2.0623 | 0.0064 |
| *Hnrnpab* | Heterogeneous nuclear ribonucleoprotein A/B | -1.2292 | 0.0416 |
| *Hsd17b7* | Hydroxysteroid (17-beta) dehydrogenase 7 | -1.5221 | 0.0480 |
| *Idi1* | Isopentenyl-diphosphate delta isomerase 1 | -2.0524 | 0.0056 |
| *Irs2* | Insulin receptor substrate 2 | 1.6998 | 0.0415 |
| *Itgal* | Integrin, alpha L | 1.3163 | 0.0339 |
| *Kif26b* | Kinesin family member 26B | -3.0480 | 0.0350 |
| *Klf9* | Kruppel-like factor 9 | 1.4918 | 0.0195 |
| *Klhdc2* | Kelch domain containing 2 | -1.1145 | 0.0375 |
| *Lars* | Leucyl-tRNA synthetase | -1.1103 | 0.0287 |
| *Lmna* | Lamin A | -1.2039 | 0.0392 |
| *LOC100359951* | | -1.3843 | 0.0415 |
| *LOC100361376* | | -1.4853 | 0.0389 |
| *LOC494499* |  | -1.5858 | 0.0441 |
| *LOC682888* |  | -1.4953 | 0.0172 |
| *Lonp2* | lon peptidase 2, peroxisomal | -1.2148 | 0.0392 |
| *Lrrc59* | Leucine rich repeat containing 59 | -1.1868 | 0.0311 |
| *Lss* | Lanosterol synthase (2,3-oxidosqualene-lanosterol cyclase) | -1.5008 | 0.0022 |
| *Maged1* | Melanoma antigen, family D, 1 | -1.1454 | 0.0420 |
| *Man1b1* | Mannosidase, alpha, class 1B, member 1 | -1.0810 | 0.0499 |
| *Mars* | Methionine-tRNA synthetase | -1.2022 | 0.0009 |
| *Mcm3ap* | Minichromosome maintenance complex component 3 associated protein | -1.1150 | 0.0263 |
| *Me1* | Malic enzyme 1, NADP(+)-dependent, cytosolic | -1.7325 | 0.0308 |
| *Med29* | Mediator complex subunit 29 | -1.2641 | 0.0351 |
| *Mertk* | c-mer proto-oncogene tyrosine kinase | 1.1888 | 0.0450 |
| *Mid1ip1* | MID1 interacting protein 1 (gastrulation specific G12 homolog (zebrafish)) | -1.5504 | 0.0262 |
| *Mocs2* | Molybdenum cofactor synthesis 2 | -1.1475 | 0.0375 |
| *Mphosph10* | M-phase phosphoprotein 10 (U3 small nucleolar ribonucleoprotein) | -1.2450 | 0.0106 |
| *Mrpl19* | Mitochondrial ribosomal protein L19 | -1.2330 | 0.0415 |
| *Mrps18b* | Mitochondrial ribosomal protein S18B | -1.3708 | 0.0262 |
| *Mrps2* | Mitochondrial ribosomal protein S2 | -1.3651 | 0.0009 |
| *Mrps34* | Mitochondrial ribosomal protein S34 | -1.3264 | 0.0365 |
| *mt-Cytb* | Mitochondrially encoded cytochrome b | -1.1483 | 0.0476 |
| *mt-Nd3* | NADH-ubiquinone oxidoreductase chain 3 | -1.1733 | 0.0262 |
| *Mug2* | Murinoglobulin 2 | 1.2841 | 0.0363 |
| *Mvd* | Mevalonate (diphospho) decarboxylase | -2.0150 | 0.0195 |
| *Mybbp1a* | MYB binding protein (P160) 1a | -1.2204 | 0.0311 |
| *Naa50* | N(alpha)-acetyltransferase 50, NatE catalytic subunit | -1.1406 | 0.0375 |
| *Nabp1* | Oligonucleotide/oligosaccharide-binding fold containing 2A | -1.3573 | 0.0255 |
| *Ndufaf4* | NADH dehydrogenase (ubiquinone) 1 alpha subcomplex, assembly factor 4 | -1.3623 | 0.0106 |
| *Nmi* | N-myc (and STAT) interactor | -1.1395 | 0.0307 |
| *Nsdhl* | NAD(P) dependent steroid dehydrogenase-like | -1.1795 | 0.0140 |
| *Nsfl1c* | NSFL1 (p97) cofactor (p47) | -1.2548 | 0.0212 |
| *Nubp2* | Nucleotide binding protein 2 | -1.2514 | 0.0434 |
| *Nutf2* | Nuclear transport factor 2 | -1.1985 | 0.0416 |
| *Oat* | Ornithine aminotransferase | 1.3563 | 0.0324 |
| *Olr1587* | Olfactory receptor 1587 | 7.9442 | 0.0415 |
| *Pam* | Peptidylglycine alpha-amidating monooxygenase | 1.3639 | 0.0324 |
| *Parp1* | Poly (ADP-ribose) polymerase 1 | -1.1740 | 0.0090 |
| *Pcbd1* | Pterin-4 alpha-carbinolamine dehydratase/dimerization cofactor of hepatocyte nuclear factor 1 alpha | -1.0616 | 0.0476 |
| *Pck1* | Phosphoenolpyruvate carboxykinase 1 (soluble) | 1.7312 | 0.0090 |
| *Pcsk9* | Proprotein convertase subtilisin/kexin type 9 | -1.3723 | 0.0324 |
| *Pdcd11* | Programmed cell death 11 | -1.1136 | 0.0195 |
| *Pfas* | Phosphoribosylformylglycinamidine synthase | 1.1648 | 0.0442 |
| *Pinx1* | PIN2/TERF1 interacting, telomerase inhibitor 1 | -1.4089 | 0.0375 |
| *Pir* | Pirin (iron-binding nuclear protein) | -1.9381 | 0.0370 |
| *Pnrc2* | Proline-rich nuclear receptor coactivator 2 | 1.0710 | 0.0480 |
| *Polr3c* | Polymerase (RNA) III (DNA directed) polypeptide C | -1.1944 | 0.0441 |
| *Ppm1a* | Protein phosphatase 1A, magnesium dependent, alpha isoform | -1.1031 | 0.0389 |
| *Ppp1r3b* | Protein phosphatase 1, regulatory subunit 3B | -1.3082 | 0.0195 |
| *Ppp2r1a* | Protein phosphatase 2, regulatory subunit A, alpha | -1.1987 | 0.0369 |
| *Ppp2r4* | Protein phosphatase 2A activator, regulatory subunit 4 | -1.2064 | 0.0476 |
| *Preb* | Prolactin regulatory element binding | -1.2240 | 0.0416 |
| *Prpf19* | PRP19/PSO4 pre-mRNA processing factor 19 homolog (S. cerevisiae) | -1.1928 | 0.0324 |
| *Prrc2a* | HLA-B associated transcript 2 | -1.1399 | 0.0470 |
| *Psat1* | Phosphoserine aminotransferase 1 | -1.7933 | 0.0450 |
| *Psma1* | Proteasome (prosome, macropain) subunit, alpha type 1 | -1.1177 | 0.0212 |
| *Psma6* | Proteasome (prosome, macropain) subunit, alpha type 6 | -1.1403 | 0.0441 |
| *Psmb5* | Proteasome (prosome, macropain) subunit, beta type 5 | -1.2150 | 0.0419 |
| *Psmb6* | Proteasome (prosome, macropain) subunit, beta type 6 | -1.2061 | 0.0307 |
| *Psmc1* | Proteasome (prosome, macropain) 26S subunit, ATPase, 1 | -1.1719 | 0.0369 |
| *Psmd5* | Proteasome (prosome, macropain) 26S subunit, non-ATPase, 5 | -1.2277 | 0.0072 |
| *Psmd7* | Proteasome (prosome, macropain) 26S subunit, non-ATPase, 7 | -1.1540 | 0.0262 |
| *Psme3* | Proteasome (prosome, macropain) activator subunit 3 | 1.6157 | 0.0171 |
| *Ptcd2* | Pentatricopeptide repeat domain 2 | -1.3706 | 0.0056 |
| *Ptges2* | Prostaglandin E synthase 2 | -1.2170 | 0.0480 |
| *Ptpre* | Protein tyrosine phosphatase, receptor type, E | ***** | 0.0056 |
| *Q5U2N5* |  | -1.2520 | 0.0283 |
| *Rad23b* | RAD23 homolog B (S. cerevisiae) | -1.1217 | 0.0115 |
| *Ranbp1* | RAN binding protein 1 | -1.2808 | 0.0338 |
| *Rars* | Arginyl-tRNA synthetase | -1.1837 | 0.0196 |
| *Rdh11* | Retinol dehydrogenase 11 (all-trans/9-cis/11-cis) | -1.2314 | 0.0056 |
| *Rnf7* | Ring finger protein 7 | -1.1817 | 0.0499 |
| *Rrm2* | Ribonucleotide reductase M2 | 2.4855 | 0.0434 |
| *Rsf1* | Remodeling and spacing factor 1 | 1.2737 | 0.0442 |
| *Rtfdc1* | Replication termination factor 2 domain containing 1 | -1.1110 | 0.0434 |
| *S1pr1* | Sphingosine-1-phosphate receptor 1 | -1.2546 | 0.0072 |
| *Sc4mol* | Methylsterol monooxygenase 1 | -1.9263 | 0.0056 |
| *Sc5dL* | Sterol-C5-desaturase (ERG3 delta-5-desaturase homolog, S. cerevisiae) | -1.2508 | 0.0056 |
| *Scly* | Selenocysteine lyase | -1.3971 | 0.0113 |
| *Scn1b* | Sodium channel, voltage-gated, type I, beta | -1.4340 | 0.0056 |
| *Sds* | Serine dehydratase | 2.4717 | 0.0441 |
| *Sds* | Serine dehydratase | 4.7055 | 0.0114 |
| *Sec24c* | SEC24 family, member C (S. cerevisiae) | -1.1098 | 0.0262 |
| *Sec61a1* | Sec61 alpha 1 subunit (S. cerevisiae) | -1.1535 | 0.0210 |
| *Sema3d* | Sema domain, immunoglobulin domain (Ig), short basic domain, secreted, (semaphorin) 3D | ***** | 0.0044 |
| *Serpine2* | Serpin peptidase inhibitor, clade E, member 2 | 1.1447 | 0.0324 |
| *Sert1 (AF077195)* | Sertoli cell protein 1 | -10.2588 | 0.0415 |
| *Sf3a1* | Splicing factor 3a, subunit 1 | -1.1882 | 0.0434 |
| *Sfrs2* | Serine/arginine-rich splicing factor 2 | -1.3264 | 0.0169 |
| *Sfrs3* | Serine/arginine-rich splicing factor 3 | -1.4130 | 0.0072 |
| *Shmt2* | Serine hydroxymethyltransferase 2 (mitochondrial) | -1.1702 | 0.0434 |
| *Sin3a* | SIN3 homolog A, transcription regulator (yeast) | 1.1627 | 0.0375 |
| *Slc25a1* | Solute carrier family 25 (mitochondrial carrier, citrate transporter), member 1 | -1.2313 | 0.0307 |
| *Slc39a7* | Solute carrier family 39 (zinc transporter), member 7 | -1.2240 | 0.0106 |
| *Sord* | Sorbitol dehydrogenase | -1.0950 | 0.0373 |
| *Spcs2* | Signal peptidase complex subunit 2 homolog (S. cerevisiae) | -1.1529 | 0.0415 |
| *Spryd3* | SPRY domain containing 3 | -1.2140 | 0.0415 |
| *Sqle* | Squalene epoxidase | -2.1774 | 0.0056 |
| *Srebf1* | Sterol regulatory element binding transcription factor 1 | -1.5895 | 0.0420 |
| *Srebf2* | Sterol regulatory element binding transcription factor 2 | -1.2412 | 0.0434 |
| *Srp68* | Signal recognition particle 68 | -1.1444 | 0.0370 |
| *Ssbp1* | Single-stranded DNA binding protein 1 | -1.2542 | 0.0369 |
| *Stbd1* | Starch binding domain 1 | -1.2458 | 0.0460 |
| *Stx5a* | Syntaxin 5A | -1.1353 | 0.0415 |
| *Tbc1d2* | TBC1 domain family, member 2 | -1.2259 | 0.0113 |
| *Tbl3* | Transducin (beta)-like 3 | -1.2918 | 0.0499 |
| *Tbrg1* | Transforming growth factor beta regulator 1 | -1.1466 | 0.0415 |
| *Tcp11l2* | T-complex 11 (mouse) like 2 | 1.3396 | 0.0499 |
| *Tgfbrap1* | Transforming growth factor, beta receptor associated protein 1 | -1.1516 | 0.0435 |
| *Tgm2* | Transglutaminase 2, C polypeptide | -1.1606 | 0.0415 |
| *Thumpd1* | THUMP domain containing 1 | -1.1537 | 0.0255 |
| *Timm17a* | Translocase of inner mitochondrial membrane 17 homolog A (yeast) | -1.2752 | 0.0415 |
| *Tlr13* | Toll-like receptor 13 | 1.6008 | 0.0480 |
| *Tmem14a* | Transmembrane protein 14A | 1.2043 | 0.0177 |
| *Tmem214* | Transmembrane protein 214 | -1.2381 | 0.0263 |
| *Tmem97* | Transmembrane protein 97 | -1.4464 | 0.0142 |
| *Tnfrsf1b* | Tumor necrosis factor receptor superfamily, member 1b | -1.1961 | 0.0402 |
| *Tsku* | Tsukushi | -2.1529 | 0.0182 |
| *Ttc27* | Tetratricopeptide repeat domain 27 | -1.1974 | 0.0113 |
| *Tuba1c* | Tubulin, alpha 1C | -1.3522 | 0.0476 |
| *Tuba4a* | Tubulin, alpha 4A | -1.6102 | 0.0098 |
| *Tubb2a* | Tubulin, beta 2A class IIa | -1.6534 | 0.0113 |
| *Tubb4b* | Tubulin, beta 4B class IVb | -1.3448 | 0.0216 |
| *Txndc9* | Thioredoxin domain containing 9 | -1.1798 | 0.0056 |
| *Txnip* | Thioredoxin interacting protein | 1.1980 | 0.0195 |
| *Txnrd1* | Thioredoxin reductase 1 | -1.2881 | 0.0365 |
| *Uba5* | Ubiquitin-like modifier activating enzyme 5 | -1.1906 | 0.0182 |
| *Ubl7* | Ubiquitin-like 7 (bone marrow stromal cell-derived) | -1.1236 | 0.0441 |
| *Ubxn4* | UBX domain protein 4 | -1.1789 | 0.0134 |
| *Unc45a* | Unc-45 homolog A (C. elegans) | -1.2830 | 0.0324 |
| *Upp2* | Uridine phosphorylase 2 | 15.3404 | 0.0202 |
| *Usp39* | Ubiquitin specific peptidase 39 | -1.1462 | 0.0415 |
| *Vars* | Valyl-tRNA synthetase | -1.2386 | 0.0369 |
| *Vcp* | Valosin-containing protein | -1.0739 | 0.0262 |
| *Vsig4* | V-set and immunoglobulin domain containing 4 | 1.1169 | 0.0324 |
| *Wbscr16* | Williams-Beuren syndrome chromosome region 16 homolog (human) | -1.2372 | 0.0307 |
| *Wbscr22* | Williams Beuren syndrome chromosome region 22 | -1.2536 | 0.0283 |
| *Wdr18* | WD repeat domain 18 | -1.3181 | 0.0182 |
| *Xpnpep1* | X-prolyl aminopeptidase (aminopeptidase P) 1, soluble | -1.1476 | 0.0350 |
| *Xrcc6* | X-ray repair complementing defective repair in Chinese hamster cells 6 | -1.1766 | 0.0212 |
| *Yars* | Tyrosyl-tRNA synthetase | -1.4044 | 0.0098 |
| *Zbtb43* | Zinc finger and BTB domain containing 43 | 1.3794 | 0.0337 |
| *Zfand2a* | Zinc finger, AN1-type domain 2A | -1.3342 | 0.0407 |
| *Zfp609* | Zinc finger protein 609 | 1.1329 | 0.0480 |
| *Zhx3* | Zinc fingers and homeoboxes 3 | 1.2429 | 0.0375 |
| *Zmynd19* | Zinc finger, MYND-type containing 19 | -1.3301 | 0.0339 |
| *Znhit6* | Zinc finger, HIT-type containing 6 | -1.2740 | 0.0476 |
| ** - Gene was solely expressed in water control. ^#^ - Gene was solely expressed in ethanol treatment.* | | | |
